# Renal Cell Carcinoma Initially Presenting as an Arteriovenous Malformation: A Case Presentation and a Review of the Literature

**DOI:** 10.1155/2013/356819

**Published:** 2013-10-23

**Authors:** Samuel Volin, Peter Steinberg, Derek Mittleider

**Affiliations:** ^1^Department of Medical Education, Tufts University School of Medicine, 145 Harrison Avenue, Boston, MA 02111, USA; ^2^Department of Urology, Beth Israel Deaconess Medical Center, Boston, MA 02215, USA; ^3^Department of Radiology, Section of Vascular and Interventional Radiology, Maine Medical Center, Portland, ME 04102, USA

## Abstract

We describe a case of a patient who presented with hematuria and was diagnosed with a renal arteriovenous malformation (AVM). Transcatheter arterial embolization subsequently was performed on this lesion multiple times. Follow-up imaging demonstrated that the AVM was masking an underlying, rapidly growing renal cell carcinoma (RCC). We describe the pathological and radiographic characteristics of AVMs and RCC. We describe the strengths and weaknesses of computed tomography (CT) and magnetic resonance imaging (MRI) to detect and characterize RCC and AVM. We recommend initial and follow-up MR imaging in patients with an AVM to establish a baseline, monitor treatment response, and survey lesions for underlying and obscured malignancy.

## 1. Introduction

Renal cell carcinoma (RCC) represents 3% of all adult malignancies and is the most common primary malignancy of the kidney (92%), followed by transitional cell carcinoma of the renal pelvis and Wilm's tumor [1, 2]. Of the five subtypes of RCC, clear-cell (75–85%) and papillary are the most common [3]. RCC is known to produce high levels of angiogenic growth factors, often giving the tumor a vascular appearance [4]. This neoplasm can have obscure clinical and radiologic features including paraneoplastic, paracrine, and vascular syndromes [5].

An arteriovenous malformation (AVM) is an aberrant vascular shunt between the arterial and venous systems due to absence of an intervening capillary bed [6]. The prevalence of AVMs is 0.04% in the general population and can be congenital or acquired. Acquired renal AVMs have been associated with renal biopsy, trauma, and malignancy [7]. Angiogenic factors from a neoplasm contribute to abnormal vascular proliferation, explaining previously described cases of RCC presenting with AVMs [5, 8, 9]. Due to the varied presentation of renal AVMs, it can be difficult to establish a radiographic diagnosis. 

Both RCC and AVM can present as hematuria and flank pain, and cross-sectional imaging studies are required to distinguish these processes [7]. A benign renal AVM may be difficult to differentiate from RCC, as the neovascularity and/or tumor-related thrombus of a RCC can mimic the radiographic features of an AVM. RCC and AVMs usually can be differentiated with appropriately performed CT or MR imaging, though angiography may be required to make a definitive diagnosis. It is necessary to follow an AVM with imaging after treatment to monitor for treatment response, progression, and obscured malignancy, as this case demonstrates. 

## 2. Case

**Figure 1:**
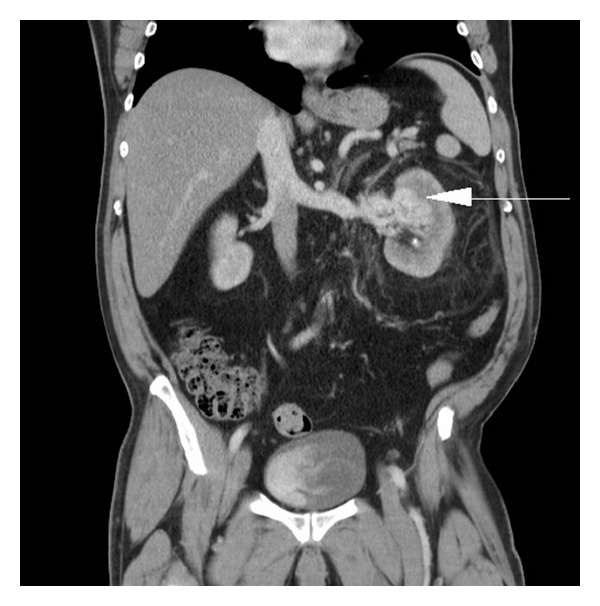
Coronal CT image showing a vascular tangle (arrow) in the left kidney.

A 63-year-old man presented to an outside hospital with two days of gross hematuria and left flank pain. He was in urinary clot retention and on continuous bladder irrigation at the outside hospital. His past surgical history was significant for an open left nephrolithotomy thirty years ago. His medical history included chronic kidney disease (GFR 87, Cr 0.88, Hb 13.3, CKD Stage G2), nephrolithiasis, and hypertension. His social history was notable for his being a practicing Jehovah's Witness who refused blood products. On physical examination the patient was noted to be obese with a left abdominal mass and a well-healed flank incision. A CT scan of the abdomen and pelvis with contrast revealed a small left renal stone and a 4.6 × 4.5 cm highly vascular mass in the anteromedial portion of the left kidney and profound urothelial thickening (Figure 1). The patient was transferred to our institution for further evaluation and management.

**Figure 2:**
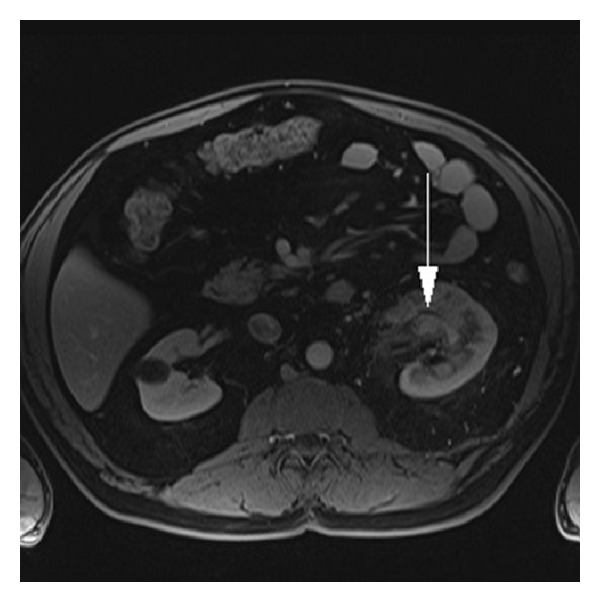
Axial MR VIBE fat saturation postcontrast depicting left-sided renal AVM (arrow). At time of imaging, the RCC could not be identified.

**Figure 3:**
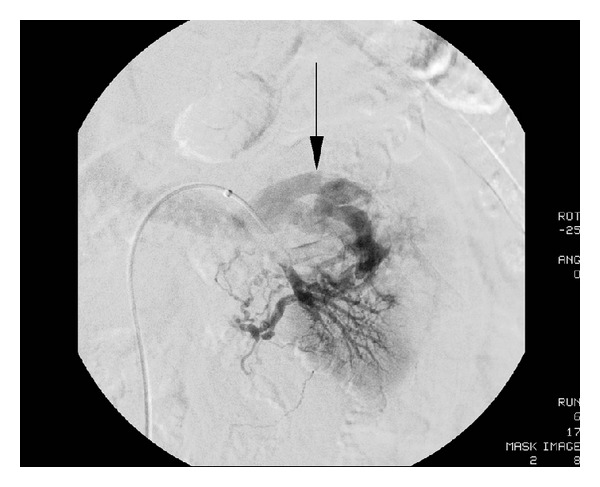
Angiography demonstrating the renal AVM with characteristic early draining vein (arrow).

**Figure 4:**
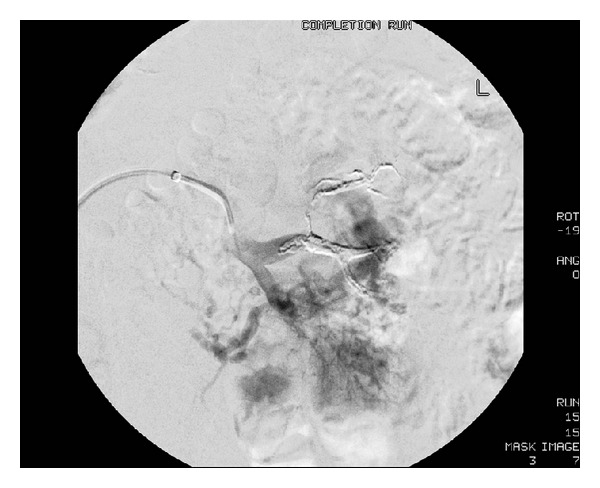
Angiography post-Onyx (ev3, Plymouth, MN) embolization of left renal AVM demonstrating decreased flow.

The patient underwent ureteroscopy, which revealed a small renal stone and no other obvious source of bleeding. A postoperative MRI demonstrated a 6 cm AVM of the left kidney (Figure 2). Interventional radiology (IR) was consulted and completed a two-stage catheter-directed embolization of the AVM with Onyx (ev3, Plymouth, MN) that resulted in 80–90% decreased flow to the AVM (Figures 3 and 4). The AVM was incompletely embolized due to the large number of feeding vessels, the total fluoroscopy time involved, concern for embolization of the entire kidney, and the need to limit the contrast dose due to renal insufficiency. His hematuria and flank pain rapidly resolved after this episode (GFR 68, Cr 1.1, Hb 12).

Four months after the initial embolization, the patient experienced recurrent gross hematuria. IR performed coil embolization of additional arterial branches supplying the renal AVM. Again, his hematuria abated after embolization (GFR 64, Cr 1.15, Hb 12).

**Figure 5:**
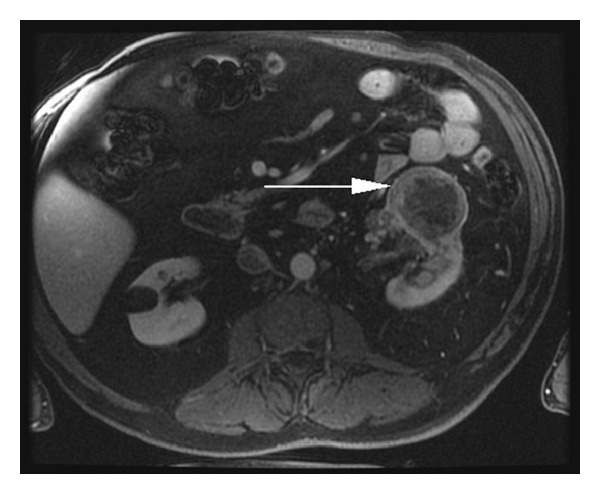
Axial dynamic MR postgadolinium LAVA sequence demonstrating RCC (arrow) in left kidney.

**Figure 6:**
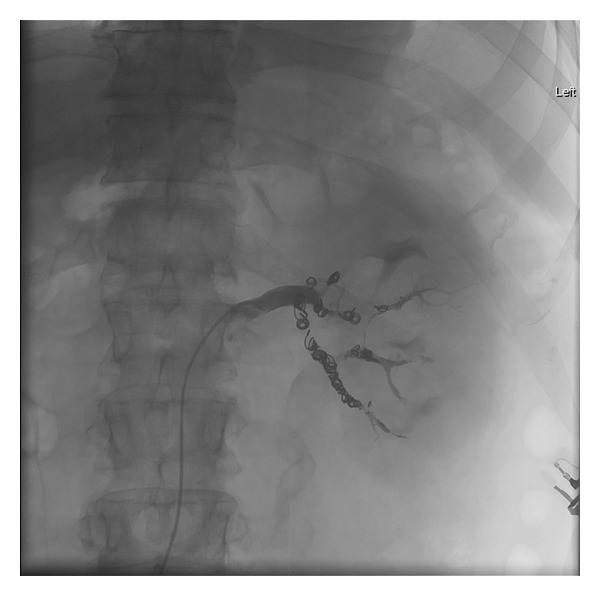
Angiography after preoperative coil embolization of left renal vein demonstrating decreased flow to left kidney.

Nine months after his initial presentation, he began to develop a chronic normocytic anemia. A full evaluation for sources of bleeding and hematologic malignancies was performed, and the only source of blood loss was felt to be a possible left renal malignancy. In the process of evaluating this anemia, a repeat MRI revealed an enhancing, heterogenous 6 cm mass that was highly concerning for a renal neoplasm arising from the left kidney (Figure 5). The AVM was present though smaller in size with margins indistinguishable from the mass lesion. Shortly after the MRI, he developed recurrent flank pain and gross hematuria and an urgent left renal angiogram with embolization of the renal artery was performed (Figure 6) (GFR 69, Cr 1.08, Hb 8.3).

The patient's case was presented at a multidisciplinary urology tumor board, and the conclusion was that radical nephrectomy was appropriate in the immediate postangiography setting. 

The following day, he underwent a challenging hand-assisted laparoscopic radical left nephrectomy (postoperative GFR 47, Cr 1.5, Hb 7.7, CKD Stage G3a). 

**Figure 7:**
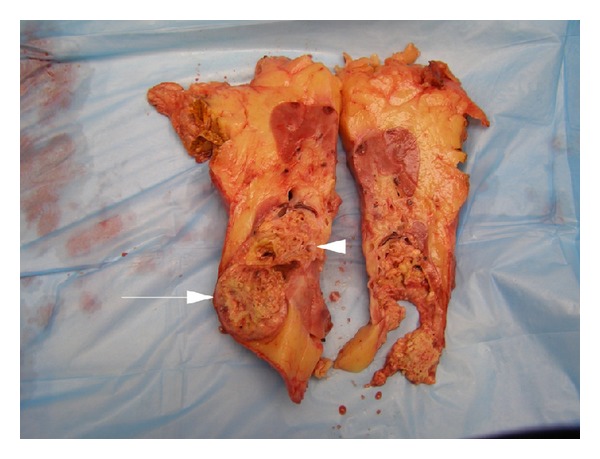
Gross image of the left kidney with RCC (arrow) and adjacent AVM (arrowhead).

Pathologic evaluation of the specimen revealed a T3aNxMx Fuhrman 2/4 7.5 cm RCC, clear cell type. The pathologist noted that prominent vessels, consistent with an AVM, were present next to the tumor (Figure 7). 

A postoperative 6-month follow-up CT scan revealed a 19 mm para-aortic lymph node. He underwent an open retroperitoneal lymph node dissection (RPLND) that revealed metastatic RCC. The patient is alive and disease-free 12 months after nephrectomy and 6 months after RPLND.

## 3. Discussion

The classic symptomatic presentations of RCC are hematuria (50–60%), abdominal pain (40%), and palpable abdominal mass (30–40%). This classic triad occurs in less than 10% of patients. Today, over half of RCC diagnoses are made after the detection of an incidental renal mass, due to the rapid growth of cross-sectional imaging [3]. Differentiation between an AVM and other renal enhancing lesions is challenging but essential in selecting appropriate management. 

Renal AVMs are rare entities, but they share a relationship with RCC [9]. The infrequency of AVMs in other malignancies suggests a unique characteristic in the local and invasive growth of RCC. RCC is a highly vascular tumor that can arise from abnormalities in the VHL gene and that leads to abnormal expression of angiogenesis-promoting growth factors such as vascular endothelial growth factor (VEGF). These angiogenic factors are crucial to the development of RCC and could explain the development of AVMs within these neoplasms [10].

**Figure 8:**
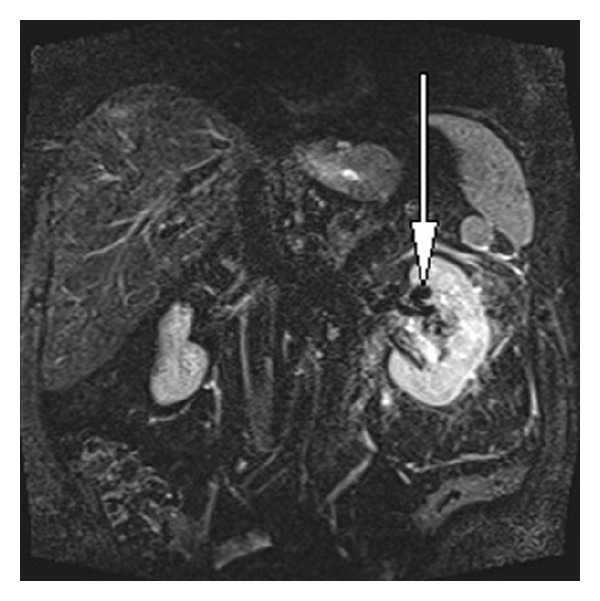
Coronal MR STIR sequence showing flow voids (arrow) within left renal AVM.

Differentiation of AVMs from RCC on cross-sectional imaging can be challenging. Varying degrees of vascular shunting are observed in both RCC and renal AVMs. MRI can help to differentiate an AVM from tumor by showing flow voids within the lesion [11] (Figure 8). Contrast-enhanced MRI of AVMs demonstrates tortuous vessels, enhancement within the cavities, and an early draining vein during the early arterial phase [12] (Figure 3). Tello et al. reported two predictors of malignancy with MRI: any enhancement on contrast-enhanced T1-weighted MR and heterogenous appearance on T2-weighted sequences (the lack of enhancement and homogeneity on T2-weighted images were indicative of benign nature). In renal tumors <2 cm in diameter, Semelka et al. found that T1-weighted fat-suppressed spin echo was superior to CT, having higher contrast resolution and better determination of presence of hemorrhage, tumor thrombus, or adenopathy [13]. In an earlier study, he showed that MRI with gadolinium-enhanced spin echo sequences had a sensitivity of 92% and was superior to CT. When combined with fat saturation sequences, the lesion detection rate with MR imaging increased to 100% [14]. 

In comparison with MRI, CT is less expensive, offers shorter examination time, and is more widely available. On CT, AVMs tend to be homogenous, enhancing structures of the same density of other vascular structures, and hypodense to parenchyma [15]. Signs of an AVM include early visualization of the ipsilateral renal vein and inferior vena cava, and the presence of numerous tortuous vessels [16].

With CT imaging, a multiphase protocol is typically utilized for the characterization of renal masses. This protocol consists of a noncontrast scan followed by contrast-enhanced scans during the corticomedullary and nephrogenic phases. This protocol allows for better detection, characterization, and more accurate staging of renal masses. The corticomedullary phase occurs between 25 and 70 seconds after contrast injection and demonstrates a brightly enhancing renal cortex and a minimally enhancing medulla. Small, hypervascular RCCs may enhance to the same degree as the cortex and may be mistaken for normal parenchyma during the corticomedullary phase. The nephrogenic phase occurs after a delay of 80 seconds and lasts up to three minutes after administration of contrast. This allows homogenous enhancement of the parenchyma, which permits distinction between normal medulla and masses. The nephrogenic phase has been shown to be most valuable in detecting and characterizing indeterminate renal masses [17].

MRI is being increasingly used as an alternative to CT. It can be advantageous when the patient has poor renal function, is allergic to contrast material, or wants to avoid ionizing radiation. Ho et al. demonstrated that on MR, a percentage enhancement threshold of 15% was able to distinguish renal cysts from solid masses on dynamic contrast-enhanced MR with 100% sensitivity and 94% specificity [18]. 

Treatment of symptomatic AVMs typically involves transcatheter arterial embolization. Embolization is minimally invasive and reduces or eliminates the vascular abnormality while maximally preserving normal parenchyma [16]. Absolute alcohol denatures proteins causing arterial spasm, sloughing of endothelium, and perivascular necrosis, typically resulting in complete and permanent occlusion. For these reasons alcohol is often the embolization agent of choice [19]. Cho et al. conducted a study on ethanol embolization of AVMs and concluded it was an effective intervention in 74% of cases [20]. Weil et al. recommend after initial treatment that AVMs be followed with digital subtraction angiography at 6 or 12 months intervals, with late follow-up at 5 years. MRA or CT angiography are less invasive alternatives for follow-up after treatment [21]. Time-resolved MR angiography (TR-MRA) can obtain fast, dynamic, contrast-enhanced vascular images that allow for the observation of the quick hemodynamic changes that occur in normal or abnormal vasculature and can be used after embolization to determine the effectiveness of the intervention [22].

Our patient initially had a multiphase CT and an MRI with contrast, both of which did not detect a RCC. A year later an MRI revealed the 6 cm enhancing mass that proved to be a RCC. As the patient had adequate initial imaging that failed to demonstrate a renal mass, this lesion grew enormously in size between the imaging evaluations. Even in retrospect, the tumor is not identifiable on the initial MRI.

The AVM was inseparable from the RCC on imaging and at surgery, and the examining pathologist noted that the two processes could be related rather than two separate entities. We believe a congenital AVM is unlikely. While a trauma-induced AVM is possible in this case, the angiogenic nature of RCC and the proximity of the lesions make it likely that this patient's AVM was secondary to or exacerbated by malignancy. 

This case demonstrates the need for serial imaging of AVMs following treatment. If this patient had been imaged at closer intervals, the RCC may have been recognized at an earlier stage prior to metastasis. Renal AVMs are difficult to distinguish from malignancy, and a high index of suspicion is needed to ensure these lesions are properly monitored.
